# Feasibility of real-time artificial intelligence-assisted anatomical structure recognition during endoscopic submucosal dissection

**DOI:** 10.1055/a-2615-8008

**Published:** 2025-06-17

**Authors:** Markus Wolfgang Scheppach, Hon Chi Yip, Yueyao Chen, Hongzheng Yang, Jianfeng Cao, Tiffany Chua, Qi Dou, Helen Mei Ling Meng, Yeung Yam, Philip W Chiu

**Affiliations:** 1 531257 Internal Medicine III - Gastroenterology, University of Augsburg Faculty of Medicine, Augsburg, Germany; 2 71024 Department of Surgery, Faculty of Medicine, The Chinese University of Hong Kong, Hong Kong SAR; 3 71024 Institute of Digestive Disease, Faculty of Medicine, The Chinese University of Hong Kong, New Territories, Hong Kong SAR; 4 26451 Department of Computer Science and Engineering, Faculty of Engineering, The Chinese University of Hong Kong, Hong Kong SAR; 5 3463 Division of Gastroenterology, Hepatology & Nutrition, Department of Medicine, College of Medicine, University of Florida, Gainesville, United States; 6 26451 Department of Systems Engineering and Engineering Management, Faculty of Engineering, The Chinese University of Hong Kong, Hong Kong SAR; 7 26451 Department of Mechanical and Automation Engineering, Faculty of Engineering, the Chinese University of Hong Kong, Hong Kong SAR

**Keywords:** Endoscopy Upper GI Tract, Precancerous conditions & cancerous lesions (displasia and cancer) stomach, Endoscopic resection (ESD, EMRc, ...), Endoscopy Lower GI Tract, Endoscopic resection (polypectomy, ESD, EMRc, ...)

## Abstract

**Background and study aims:**

Endoscopic submucosal dissection (ESD) is a challenging minimally invasive resection technique with a long training period and relevant operator-dependent complications. Real-time artificial intelligence (AI) orientation support may improve safety and intervention speed.

**Methods:**

A total of 1011 endoscopic still images from 30 ESDs were annotated for relevant anatomical structures and used for training of a deep learning algorithm. After internal and external validation, this algorithm was applied to 12 ESDs performed by either one expert or one novice in ESD using an in vivo porcine model.

**Results:**

External validation yielded mean Dice Scores of 88%, 60%, 58%, and 92% for background, submucosal layer, submucosal blood vessels, and muscle layer, respectively. The system was successfully applied during all 12 ESDs. All resections were completed en bloc and without complications.

**Conclusions:**

In this proof-of-concept study, feasibility of a real-time AI algorithm for anatomical structure delineation and orientation support during ESD was evaluated. The application proved safe and appropriate for routine procedures in humans. Further studies are needed to elucidate a potential clinical benefit of this new technology.

## Introduction

Endoscopic submucosal dissection (ESD) is a modern minimally invasive technique for resection of large superficial neoplasia of the gastrointestinal tract 1 . The method is technically challenging and requires a long training period. Complications such as bleeding and perforation are caused by inadvertent dissection through submucosal blood vessels and the muscularis propria, especially when visualization of anatomical structures and exposure of the submucosal cutting plane are insufficient. The need for trainee support in the form of observation, expert supervision as well as animal training is promoted in international consensus statements 2 . Artificial Intelligence-assisted clinical decision support solutions (AI-CDSS) for colonic polyp detection have been certified by international authorities 3 . detection and delineation of neoplasia in the upper gastrointestinal tract is the focus of current research 4 5 6 . The expected role of AI in endoscopy is predominantly one of standardization and quality improvement of diagnostic procedures 7 . However, research data on AI support during surgery 8 show that interventions may also benefit from this new technology. Preliminary research into structure identification during third-space endoscopy showed promising results 9 . Therefore, we aimed to develop an algorithm for detection and delineation of relevant anatomical structures during ESD and subsequent testing in a real-life setting.

## Methods

### Objectives and study design

The primary endpoint of this study was feasibility of application of a newly developed algorithm for intraprocedure structure identification during porcine ESD procedures. The design was a one-arm exploratory study. Feasibility was defined as continuous technically successful application during resection. Secondary endpoints included the user experience, Dice scores, and pixel accuracies of the algorithm in an internal and external validation, as well as lesion and procedure characteristics during the porcine ESDs. User experience was measured in a binary way (appropriate, score 1, not appropriate, score 0).

### Algorithm development

Thirty full-length videos of ESD procedures (2 esophageal, 26 gastric, 2 colorectal) were extracted from the database of the Prince of Wales Hospital, Hong Kong, SAR. Training data were generated using GIF-HQ290 gastrosocopes and EVIS Lucera elite processors (Olympus, Tokyo, Japan). Subsequently 1011 frames were selected for training. The categories “submucosal layer,” “muscle layer,” “submucosal blood vessel,” and “background” were delineated in each training image by an expert in ESD (> 500 procedures in human ESD). This data set was used to training a deep learning algorithm using a combination of a U-Net and transformer network. The model employed a hybrid CNN-transformer architecture to leverage both detailed localization information and global context. The model was trained and evaluated on an HP Z4 G4 workstation powered by an Intel(R) Xeon(R) W-2223 CPU operating at 3.60 GHz, featuring four physical cores and supporting hyper-threading to provide eight logical processors, in conjunction with a single Nvidia 3090 GPU equipped with 24 GB of video random access memory. All implementations were carried out using PyTorch 1.13.1 with CUDA 11.7 and Python 3.7. For pre-training the base model, the Adam optimizer with an initial learning rate of 1e-4 was employed, training the model for 10 epochs with a batch size of four. During the training process, 20% of the dataset was allocated as a dedicated test set, while the remaining 80% was split further into 80% for training and 20% for validation. Before processing by the model, video data are preprocessed by resizing to 256 × 256 resolution and normalized to zero mean and unit variance to ensure consistency across input features. The resulting algorithm was evaluated for Dice score and pixel accuracy in an internal cross-validation, as well as an external validation (220 images, 4 ESD cases, 2 esophageal, 1 gastric, 1 colorectal) obtained from Augsburg University Hospital, Augsburg, Germany. Test data were generated using GIF-EZ1500 gastroscopes with EVIS X1 processors (Olympus, Tokyo, Japan).

### Porcine model study

Demonstration video of an ESD procedure with (left side) and without (right side) AI overlay. The green semitransparent overlay indicates the muscle layer. The yellow border tracing indicates the submucosal layer. The red semitransparent overlay indicates the submucosal blood vessels.Video 1

**Fig. 1:**
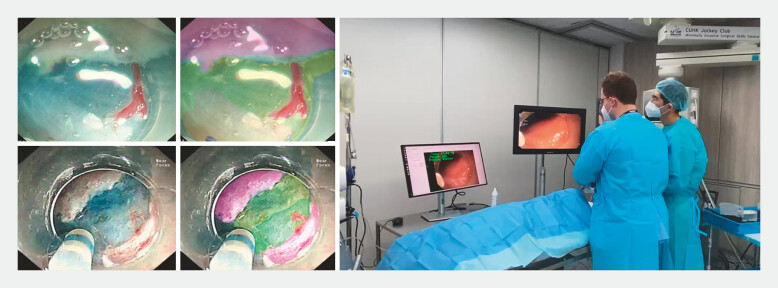
Images from the animal trial. Far left: Two endoscopic images during submucosal dissection. Left: The same images with AI prediction in a semitransparent colored overlay. Mucosa (background) is in pink, the submucosal layer is in yellow, the muscle layer is in green, and the blood vessels are in red. Right: Photo of the trial setup with the regular endoscopic screen on the right and the screen showing the AI picture in real time on the left.

An in vivo porcine model was then constructed using two live pigs. Herein, one expert and one novice in ESD (no experience in human ESD) performed esophageal, gastric, and rectal ESDs with real-time support from the AI system. For this purpose, a real-time endoscopic image was transferred to an AI station with a separate monitor equal in size and resolution to the conventional monitor, and the endoscopic image was shown continuously with real-time segmentation of the algorithm ( Fig. 1 , Video 1 ). Standard ESDs were performed for 12 lesions with a target size of 2 cm without use of traction techniques. In all procedures, DualKnife J (Olympus Medical Corporations, Tokyo, Japan) was used. Procedure times, en bloc resection rates, and intraprocedure complications such as profuse bleeding and transmural perforation were recorded. Bleeding was graded according to severity (Grade 1: Relevant oozing bleeding that requires the endoscopist to intervene, hemostasis successful by coagulation with the tip of the knife; Grade 2: Relevant oozing or spurting bleeding that rapidly covers the resection plane and causes impaired view, hemostasis successful by coagulation with the tip of the knife and/or hemostatic forceps and/or clipping; Grade 3: Fulminant bleeding causing termination and failure of the procedure or requiring hemostatic measures for more than 5 minutes). Perforation was defined as a full-thickness perforation with visibility of underlying tissue outside of the muscle layer such as the serosa, adventitia, peritoneum or mediastinum. Ninety-five percent confidence intervals (CIs) are shown in brackets. Institutional review board approval for the study was obtained from the Animal Experimentation Ethics Committee (AEEC), the Chinese University of Hong Kong (Ref. No. 22–145-MIS).

## Results

The AI system was continuously and successfully applied during all 12 ESD procedures ( Table 1 ). Feedback from both endoscopists showed an appropriate function of the algorithm for each ESD procedure by both interventionists. Internal cross-validation showed mean Dice scores of 91% (95% CI 90%-93%), 77% (95% CI 74%-80%), 60% (95% CI 54%-67%), 82% (95% CI 78–85), and mean pixel accuracies of 9% (95% CI 94%-96%), 73% (95% CI 70%-76), 60% (95%CI 53%-66%), 82% (95% CI 79%-86%) for background, submucosal layer, submucosal blood vessels, and muscle layer, respectively. On the external dataset, Dice scores of 90% (95% CI 89%-91%), 68% (95% CI 65%-71%), 56% (95% CI 49%-62%), and 92% (95% CI 89%-96%) and pixel accuracies of 95% (95% CI 94%-96%), 63% (95% CI 61%-67%, 55% (95% CI 49%-62%), and 92% (95% CI 89%-95%) were calculated for the same classes. The algorithm generated real-time annotation of the trained anatomical structures with a latency of 0.04 seconds at a frame rate of 25 frames per second. All ESDs were successfully performed with a 100% en bloc resection rate and no major intraprocedure complications. Mean resection time per specimen size was 14.8 minutes (95% CI 9.8–19.8). Bleeding occurred 4.8 times per ESD on average (95% CI 3.4–6.1) (grade 1: 4.2, 95% CI 3.0–5.3); grade 2: 0.6, 95% CI 0.2–1.0); grade 3: 0 (95%CI 0–0). The rate of bleeding per specimen size was 2.0 per cm (95% CI 1.1–2.8). There were no full-thickness perforations.

**Table:** **Table 1** Results from the in vivo porcine model AI application trial.

	Expert	Novice	Overall
Resection characteristics			
Esophagus	2	2	4
Stomach	3	2	5
Rectum	2	1	3
Overall	7	5	12
Mean resection time in minutes:	33	48	39
Feasibility test			
Technical feasibility of the AI algorithm [%]	100	100	100
User experience for appropriate function	7	5	12
Specimens			
Number of en bloc resections	7	5	12
Number of specimen perforations	0	0	0
Mean specimen size in cm	2.9	2.6	2.8
Complications			
Bleeding overall	4.9	4.6	4.8
Bleeding Grade I	4.3	4.0	4.2
Bleeding Grade II	0.6	0.6	0.6
Bleeding Grade III	0	0	0
Perforation	0	0	0

## Discussion

In this preliminary study, a novel AI algorithm for real-time delineation of anatomical structures during ESD was developed. Technical feasibility for usage in a porcine model could be demonstrated for the expert and novice endoscopist in a real-life setting. Thus, the primary goal of this study was achieved, that is, proof of concept of real-time application of an AI-CDSS for ESD. User experience evaluation showed appropriate usability of the algorithm. The in vivo porcine model is considered the most appropriate experimental model for ESD procedures 10 . Results from the internal validation were deemed satisfactory, whereas reduced performance for certain classes was measured in external validation. The decrease in Dice scores and pixel accuracies for the classes submucosa and submucosal blood vessel on the external dataset show the as-yet-limited robustness of the algorithm when confronted with images from different endoscopy systems. To reduce possible overfitting, different image modalities from different endoscopes will be included in the training data for future versions of the algorithm. Feasibility testing was conducted with a limited number of animal procedures and interventionists. However, in this early stage of development of a new technology, a test with a limited number of animal subjects was deemed ethically appropriate, while still large enough for a meaningful result. Further development of the algorithm with more training data as well as more concise preclinical evaluation of performance should precede human trials.

## Conclusions

In conclusion, although real-time decision support during ESD seems viable and may offer valuable assistance especially for trainees, confirmatory studies are needed in order to corroborate these hypotheses.
